# The effects of video-based and blended learning on medication dosage calculation skills of paramedic students: A randomized, quasi-experimental study

**DOI:** 10.1097/MD.0000000000043651

**Published:** 2025-08-01

**Authors:** Leyla Baran, Huri Öztürk

**Affiliations:** a Faculty of Health Sciences, Mardin Artuklu University, Mardin, Türkiye; b Vocational School of Health Services, Izmir Democracy University, Izmir, Türkiye.

**Keywords:** blended learning, medication dosage calculation, online education, paramedics, video-based learning

## Abstract

**Background:**

Medication dosage calculation (MDC) is a vital component of clinical competence for healthcare providers, particularly in emergency medical services, where miscalculations can lead to life-threatening outcomes. This study aimed to compare and evaluate the effects of video-based learning (VBL) and blended learning (BL) on the MDC skills of paramedic students, with the hypothesis that BL would result in higher posttest MDC performance compared to VBL.

**Methods:**

A randomized quasi-experimental design was used. A total of 151 paramedic students were randomly assigned to 2 groups. Both groups received a 2-week e-learning course that included video-based content and task-based exercises. Additionally, the BL group received an 8-hour classroom-based training session during the same 2-week period, which was scheduled as a single full-day session on the third day of the first week. Following the intervention, both groups completed a posttest assessing their MDC skills.

**Results:**

The BL group achieved significantly higher posttest scores (82.19 ± 13.74) compared to the VBL group (72.24 ± 21.87), with the difference being statistically significant (*P* = .001).

**Conclusion:**

While both methods were effective in enhancing MDC skills, BL was more effective. E-learning materials require further development to function as a fully independent instructional approach.

## 1. Introduction

Medication dosage calculation (MDC) is a critical responsibility for all healthcare professionals involved in medication administration. This process begins with a medication order and requires the accurate and precise determination of dosage to ensure patient safety.^[1]^ This skill goes beyond basic arithmetic and falls within the broader domain of numerical literacy – the ability to select appropriate mathematical tools, apply them accurately, and assess the contextual relevance of the results.^[2,3]^

MDC comprises a variety of sub-skills, including calculations for tablet, liquid, and intravenous infusion doses. Effective dosage calculation requires both conceptual and arithmetical competence.^[4]^ Conceptual competence refers to the ability to accurately interpret medication labels, dosage units, and prescriptions. Arithmetical competence involves performing unit conversions, working with fractions, and executing decimal operations.^[5]^

Although MDC is often associated with nursing education, paramedics working in emergency medical services also have a strong need for these skills. In fact, out-of-hospital environments introduce additional complexity to dosage calculations due to stress, distractions, and hazardous environmental factors.^[6–8]^ Such conditions may negatively affect paramedics’ decision-making and safe medication administration abilities.^[9]^ While most dosage errors are attributed to these stressful conditions, some studies have shown that paramedics struggle with basic mathematical skills even in controlled classroom settings.^[10–12]^ For instance, one study found that the correct dosage calculation rate was only 68%, and that experienced paramedics made more errors than less experienced ones.^[13]^ A systematic review by Walker and colleagues (2023) confirmed that these challenges persist in prehospital settings.^[9]^

These findings indicate that paramedics must possess strong mathematical and conceptual skills. Such competencies are essential not only for professional success but also for patient safety.^[7]^ However, effective strategies to enhance these skills remain limited in the current literatüre.^[14]^

The effectiveness of instructional methods for MDC training in undergraduate health programs remains a topic of debate.^[1,15]^ While there is a common perception that online methods are insufficient for developing clinical skills^[16]^ some studies have demonstrated the effectiveness of video-based learning (VBL) and blended learning (BL) in enhancing MDC skills.^[17–19]^ Conversely, other research has reported no significant difference between technology-assisted and traditional teaching methods.^[20,21]^ Nevertheless, the overall trend indicates that traditional paper-based or blackboard-centered instruction is no longer adequate.

The COVID-19 pandemic accelerated the shift toward digitalization; with the disruption of face-to-face education, online learning became inevitable.^[22]^ This transformation not only increased the demand for digital resources but also highlighted the limitations of conventional instructional methods.^[16]^

Accordingly, this study aims to compare the effectiveness of 2 different instructional methods in improving MDC skills among Emergency Medical Technician-Paramedic (EMTP) students. The methods compared are fully online VBL and BL. The study seeks to determine whether VBL alone is sufficient or whether it serves only as a supplementary instructional approach. Thus, the study aims to provide evidence-based insights into the most effective teaching strategies for developing MDC skills among EMTP students.

The study hypotheses were stated as follows:

**H**_**1**_: Both VBL and BL have a positive impact on the development of MDC skills among EMTP students.
**H**_**2**_: EMTP students who receive BL demonstrate significantly higher posttest MDC performance compared to those who receive VBL.

## 2. Materials and methods

### 2.1. Study design

**Figure 1.**
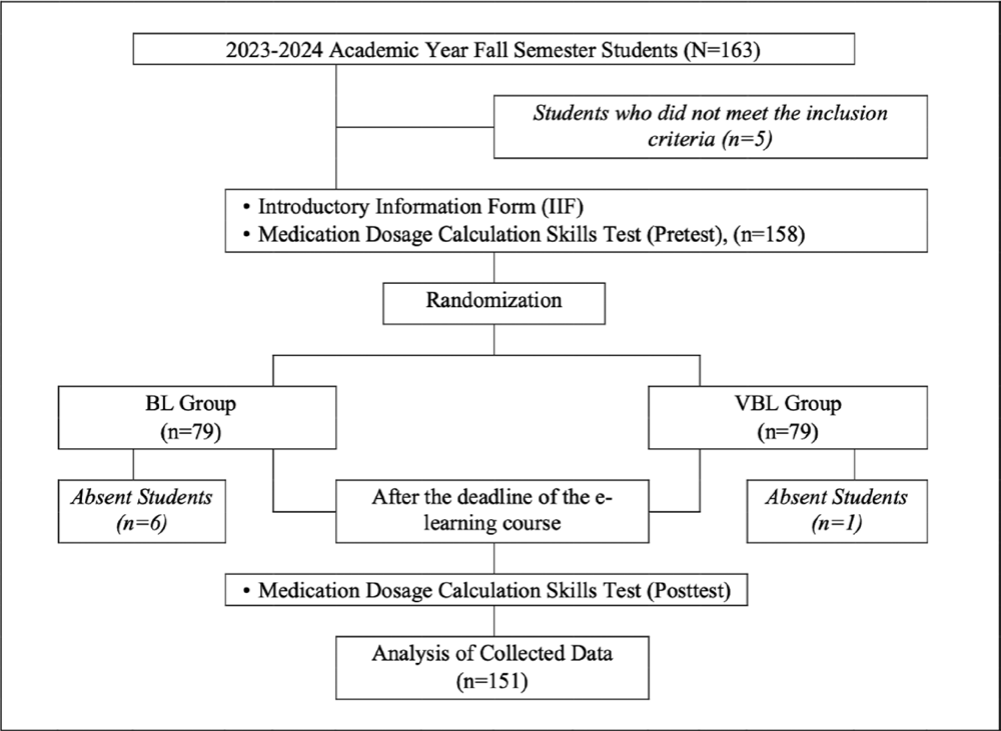
Flow diagram of the study.

This study was conducted using a 2-group pretest - posttest design. Both groups had an e-learning course on MDC, which lasted 2 weeks, and the BL group received an additional 8-hour training in the classroom. The 8-hour classroom-based training for the BL group was conducted as a single full-day session on the third day of the 2-week intervention period. The students were assigned to the groups in accordance with randomization (Fig. 1).

After the study data were collected, the VBL group was given theoretical training in the classroom for 8 hours so as to avert any ethical problems.

### 2.2. Settings and participants

The population of this study consisted of EMTP students (N = 163) studying at a university in the Southeastern Anatolia Region of Türkiye during the fall semester of the 2023/2024 academic year. No sampling method was used; the study aimed to reach the entire population.

#### 2.2.1. Inclusion criteria

Being a student in the EMTP department and attending classes regularly.

#### 2.2.1. Exclusion criteria

Being a student in a different department that teaches MDC or having prior education/training on MDC, having internet access problems and providing incomplete data.

### 2.3. Randomization

We acknowledge that students’ learning styles and numerical competency levels may act as confounding factors in MDC skills. While these variables were considered for randomization, they present inherent biases and practical limitations. For example, students may be unaware of their learning style, and self-reported numerical competency may not accurately reflect their actual proficiency.

Previous studies that employed randomization to assign students into 2 groups primarily based their methods on grade point average (GPA) or simple randomization.^[19,20,23]^ However, research has shown that students with higher GPAs tend to achieve higher MDC test scores.^[19]^ To control this effect, we ranked 158 students based on their GPA and systematically assigned them to 2 groups: those with odd-numbered rankings were placed in the BL group, while those with even-numbered rankings were allocated to the VBL group.

### 2.4. Data collection tool

“Introductory Information Form” and “Medication Dosage Calculation Skills Test (MDCST)” were used to collect the data.

#### 2.4.1. Introductory Information Form

Created by the researchers based on the relevant literature, the form consisted of 7 open- and closed-ended questions about participants’ sociodemographic data such as GPA, age, gender, type of high school from which the students had graduated, individual assessment of the level of mathematical skills, use of a calculator in mathematical operations, and prior training on MDC.

#### 2.4.2. Medication Dosage Calculation Skills Test

Adapted from Öztürk and Güneş (2023) with their permission.^[19]^ The test consisted of 20 questions under 5 basic topics determined in line with the learning objectives in an attempt to assess MDC skills of the students. The topics were “Units of Measure and Conversions,” “Tablets and Capsules,” “Oral Suspensions,” “Injections” and “Infusion Treatments.”

The test was reconstructed to create 2 isomorphic versions by modifying the numerical values in all questions while preserving their original structure. To maintain consistency in difficulty levels, fractional and whole numbers were used in a manner similar to the original test, in accordance with recommendations from the literatüre.^[24]^ This approach ensured that the validity, quality, and difficulty of both tests remained consistent and that they assessed the same skills as the original test. Additionally, it facilitated reassessment after the interventions while minimizing the likelihood of students recalling the original questions.

### 2.5. Data collection

Data collection process was implemented as follows:

***Stage 1:*** The students who had enrolled in the EMTP programme (N = 163) were informed about the purpose of the study in the classroom environment and those who wanted to participate and met the inclusion criteria were identified.
***Stage 2:*** Consent of the students who agreed to participate and met the inclusion criteria (n = 158) was obtained. Then, the Introductory Information Form and the pre-MDCST were administered.
***Stage 3***: All the participants (n = 158) were ranked according to their GPA and then distributed to the groups as follows: 1st, 3rd, 5th, …, 157th students (odd numbers) in the ranking were classified into the BL group and 2nd, 4th, 6th, …, 158th students (even numbers) into the VBL group.
***Stage 4:*** Both groups received a 2-week e-learning course on MDC. Additionally, the BL group had a traditional classroom-based course for 8 hours. In this stage, 7 students provided incomplete data.
***Stage 5:*** The e-learning course remained open for 2 weeks, and after the period ended, the post-MDCST assessment was administered to all participants immediately to ensure consistent timing for both groups (n = 151).
***Stage 6:*** The collected data were analyzed.
***Stage 7:*** During the data collection process, while the BL group received 8 hours of traditional classroom-based training, the VBL group was unable to participate in the same training. To ensure equality and avoid any gap in the educational experience of the VBL group, an additional 8 hours of theoretical training was provided to them after the completion of the data analysis phase. This training was separate from the data collection process and was conducted solely to ensure that both groups had equal training opportunities.

### 2.6. Data analysis

The research data were analyzed with the Package for Social Sciences, Version 26.0 (SPSS Inc., Chicago).

- Numbers, percentage distributions, means, standard deviations as well as minimum and maximum values related to the participants’ demographic data were presented in Table 1.
- The answers to the pre- and post-MDCST were coded as correct and incorrect and each correct answer was awarded 5 points. The results were evaluated out of 100 points.
- The normality of the distribution of the data was evaluated by Kolmogorov-Smirnov and Shapiro–Wilk tests.
- Independent group *t* test was used to determine whether the pretest and posttest scores showing normal distribution differed according to the groups.
- The statistical significance level was accepted as *P* < .05.

**Table 1:** Demographic characteristics of the students.

Demographic Characteristics	BL Group		VBL Group			Total		
	n	$\bar{X}\pm \;SD$ (Min–Max)	n	$\bar{X}\pm \;SD$ (Min–Max)		n	$\bar{X}\pm \;SD$ (Min–Max)	
Age	73	20.85 ± 2.01 (18–28)	78	20.58 ± 1.91 (18–30)		151	20.71 ± 1.96 (18–30)	
GPA	73	71.17 ± 7.34 (54.00–88.00)	78	72.88 ± 6.83 (54.50–88.00)		151	72.05 ± 7.10 (54.00–88.00)	
Individual assessment of the level of mathematical skills	73	3.32 ± 0.78 (1–5)	78	3.15 ± 0.94 (1–5)		151	3.23 ± 0.86 (1–5)	

Demographic characteristics			n	%	n	%	n	%
Gender	Female		43	58.9	46	59.0	89	58.9
	Male		30	41.1	32	41.0	62	41.1
Type of high school	General high school		6	8.2	3	3.8	9	6.0
	Anatolian high school		43	58.9	50	64.1	93	61.6
	Science high school		1	1.4	--	--	1	0.7
	Private high school		5	6.8	6	7.7	11	7.3
	Other high school		18	24.7	19	24.4	37	24.5
Use of a calculator in mathematical operations	Yes		1	14	5	6.4	6	4.0
	Sometimes		36	49.3	45	57.7	81	53.6
	No		36	49.3	28	35.9	64	42.6
To receive training on MDC while continuing the current program	Yes		9	12.3	5	6.4	14	9.3
	No		64	87.7	73	93.6	137	90.7
Total			73	100	78	100	151	100

## 3. Results

A final number of 151 students (participation rate = 93%) provided complete data, of whom 73 were in the BL group and 78 in the VBL group. The students in the BL group had a mean age of 20.85 ± 2.01 (18–28) while it was 20.58 ± 1.91 (18–30) years in the VBL group. In both groups, the majority of the students (58.9% in the BL group, 64.1% in the VBL group) had graduated from an Anatolian High School.

Most of them either “agreed” or “strongly agreed” that they were well-skilled in mathematical calculations (Table 1). There was no significant difference between GPA levels of 2 groups (*t* = −1.48, *P* = .140).

The independent samples *t* test was used to determine whether the normally distributed pretest and posttest scores differed between the groups, as shown in Table 2. According to pre- and post-MDCST scores, both BL and VBL techniques helped EMTP students improve their MDC skills. The mean score of the pretest was 31.54 ± 19.92 in the BL group and 26.12 ± 16.18 in the VBL group whereas for the posttest, it was 82.19 ± 13.74 in the BL group and 72.24 ± 21.87 in the VBL group. The difference between posttest results of the 2 groups was statistically significant (*P* = .001).

**Table 2:** Difference between pretest and posttest score averages of the groups.

Test	Group	Min score	Max score	$\bar{X}\pm \;SD$	*t*	*P*
Pretest	BL	00.00	75.00	31.54 ± 19.92	1.827	.070
	VBL	00.00	75.00	26.12 ± 16.18		
Posttest	BL	45.00	100.00	82.19 ± 13.74	3.369	**.001**
	VBL	25.00	100.00	72.24 ± 21.87		

All comparisons were made using the independent samples *t* test. Bold values indicate statistical significance at *P* < .05.

BL = blended learning, VBL = video-based learning.

The independent samples *t* test was used to determine whether the normally distributed pretest and posttest scores differed between the groups, as shown in Table 3. There were statistically significant differences between the mean scores of the groups in 3 of 5 learning areas of MDC: SI Units and conversions, tablets and capsules, and infusion treatments. In each learning area, the BL group had higher posttest scores than the VBL group, leading to the inference that BL is more effective than VBL alone in terms of gaining MDC competency in paramedical discipline.

**Table 3:** Difference between pretest and posttest score averages of the groups based on 5 MDC learning areas.

Topic	Group	BL	VBL	MD (95%CI)	*t*	*P*
		$\bar{X}\pm \;SD$	$\bar{X}\pm \;SD$			
SI Units and conversions (3 questions)	Pretest	6.02** ± **5.71	5.64** ± **4.92	0.38	0.444	.658
	Posttest	13.35** ± **2.50	12.11** ± **3.90	1.24	2.337	**.021**
Tablets and Capsules (3 questions)	Pretest	7.12** ± **4.63	5.06** ± **3.64	2.05	1.92	.057
	Posttest	10.54** ± **2.83	9.42** ± **4.26	1.12	3.02	**.003**
Oral Suspensions (3 questions)	Pretest	5.45** ± **4.23	5.16** ± **4.49	0.28	0.401	.689
	Posttest	11.16** ± **4.04	10.19** ± **4.29	0.97	1.429	.155
Injections (8 questions)	Pretest	11.30** ± **10.07	8.65** ± **9.45	2.64	1.666	.098
	Posttest	34.31** ± **7.65	31.66** ± **11.03	2.64	1.722	.087
Infusion Treatments (3 questions)	Pretest	1.64** ± **3.01	1.60** ± **2.96	0.04	0.085	.932
	Posttest	12.80** ± **4.24	8.84** ± **6.28	3.96	4.562	**.000**
**Total**	Pretest	31.54** ± **19.92	26.12** ± **16.18	5.41	1.827	.070
	Posttest	82.19** ± **13.74	72.24** ± **21.87	2.95	3.369	**.001**

All comparisons were made using the independent samples *t* test. Bold values indicate statistical significance at *P* < .05.

BL = blended learning, MDC = medication dosage calculation, VBL = video-based learning.

## 4. Discussion

To our knowledge, this is the first study to explore the impact of a learning intervention on the MDC performance of EMTP students in the Turkish context. Accordingly, we framed our discussion around the effects of these 2 educational approaches on students’ MDC skills and compared our findings to those from different contexts such as paramedic students in other countries, various student groups in Türkiye, and countries with similar cultural backgrounds.

Since students were allowed to use a calculator in pre- and post-MDCST, we presumed that no “computational errors” occurred. However, given the fact that most pretest errors are conceptual in natüre,^[25]^ it might be inferred that students have fundamental deficiencies in their mathematical understanding. In this manner, this study further corroborates existing evidence of poor mathematical and numeracy skills among paramedic students.^[5,10,13,26]^

Our findings supported H_1_ by demonstrating that both methods were highly beneficial to reducing conceptual errors made in the pretest, which was also underpinned by previous studies.^[25,27]^ However, some “arithmetical errors” associated with the basic knowledge to create and operate an equation and “conceptual errors” arising from a lack of understanding MDC concept were still observed in the posttest results of both groups. A possible explanation might be that students’ numerical abilities were negatively and indirectly influenced by math anxiety.^[14]^

To determine whether paramedic students experienced greater math anxiety than nursing students, Öztürk and Güneş (2023) provided an appropriate basis to compare MDC performance of these 2 groups.^[19]^ They employed the same learning materials with similar test questions for both paramedic and nursing students within the same context so as to ensure comparability and found that compared to paramedic students, nursing students had superior performance in the posttest across all the learning areas with a mean score of 92.5 ± 8.52. This comparison suggests that paramedic students may experience higher levels of math anxiety than nursing students or MDC curriculum in the paramedic program does not sufficiently enhance students’ mathematical abilities. Therefore, revising the paramedic curriculum could boost students’ self-confidence and help them overcome math anxiety related to the MDC.

Previous studies reported that although nursing students struggled with MDC in the classroom settings, they were able to easily grasp the main points of appropriate dosing through clinical experience.^[28–30]^ This might be related to the environmental cues in clinical practice which traditional learning and assessment methods cannot offer, such as using a drip chamber to determine how many drops are needed to administer.^[24]^ From this perspective, our findings implicated that the BL approach provided better learning outcomes regarding MDCST performance compared to using e-learning materials alone. This supports the view that combining classroom-based instruction with e-learning materials simulating clinical environments enhances learning outcomes. Moreover, it supports our second hypothesis (H2), which confirmed a statistically significant difference in MDC test performance in favor of the BL group.

However, it seems that even our contextualized and visualized e-learning material failed to provide such a level of practice environment experience for paramedic students. Still, this does not account for the instances where practicing nurses and paramedics have been shown to perform poorly on MDC tests.^[13,31]^ Thus, further research is necessary to explore whether this issue arises from an inability to translate the practical application of MDC into written assessments or there is a more fundamental problem at play regarding the educational strategies.

## 5. Limitations

This study was insightful in presenting areas of concern within paramedic education that need to be addressed to ensure MDC competency and maintain patient safety. However, performance on written tests in classroom settings may not always predict success in practical environments. Hence, further studies are required to assess whether the observed improvement in performance leads to a reduction in MDC errors in clinical practice.

The posttest results of this study demonstrated an improvement in skills; however, the long-term impact of these teaching methods remains uncertain. Since the posttest was administered immediately after the 2-week intervention period, future studies should investigate whether the observed improvement in skills is retained over time. There could be an additional data collection phase to determine whether this improvement in skills remains consistent after the courses or continuous exposure to such practices is necessary for its retention.

Lastly, the study was limited to a single institution, which may affect its generalizability to paramedic students at other universities.

## 6. Conclusion

Involving online materials and revising teaching strategies in the paramedic curriculum can enhance students’ MDC abilities. However, educators in paramedicine should also focus on students’ self-confidence undermined by math anxiety. In addition, since no error is acceptable for patient safety, educators should consider integrating contextualized online materials and elements from real or simulated clinical practice into traditional education to ensure a full success.

## Acknowledgments

We would like to acknowledge the students who participated in this study.

## Author contributions

**Conceptualization:** Leyla Baran, Huri Öztürk.

**Data curation:** Leyla Baran, Huri Öztürk.

**Formal analysis:** Leyla Baran, Huri Öztürk.

**Investigation:** Leyla Baran.

**Project administration:** Leyla Baran.

**Writing – original draft:** Leyla Baran, Huri Öztürk.

**Writing – review & editing:** Leyla Baran, Huri Öztürk.
